# Lyme disease control 2.0: Advances and opportunities coming with Lyme disease vaccine VLA15

**DOI:** 10.1371/journal.ppat.1013747

**Published:** 2025-12-15

**Authors:** Ondrej Hajdusek, Kalvis Brangulis, Luise Robbertse, Rajesh Ghosh, Dino Di Carlo, Jan Perner

**Affiliations:** 1 Institute of Parasitology, Biology Centre, Czech Academy of Sciences, Ceske Budejovice, Czech Republic; 2 Latvian Biomedical Research and Study Centre, Riga, Latvia; 3 Bioengineering Department, University of California, Los Angeles, California, United States of America; Tufts Univ School of Medicine, UNITED STATES OF AMERICA

## 1. A renewed opportunity for a Lyme disease vaccine

**Fig 1:**
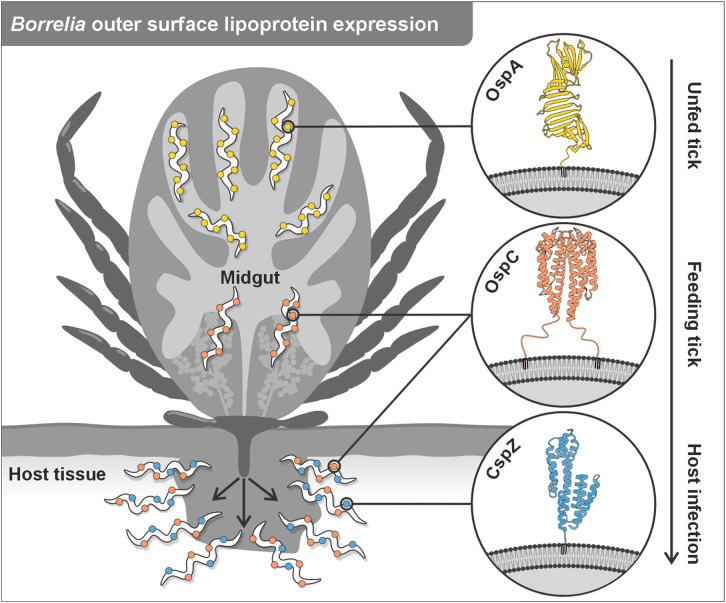
Temporal and spatial regulation of *Borrelia* outer surface proteins expression during tick-to-host transmission. During tick feeding, *Borrelia* alters the expression of its outer surface proteins [4] in response to environmental cues. OspA (yellow) is highly expressed in the midgut of unfed ticks, where it is thought to mediate adherence to the midgut epithelium. As the tick begins feeding, OspA is downregulated, while OspC (orange) is upregulated to facilitate early mammalian invasion. During systemic infection in the vertebrate host, CspZ (blue) becomes expressed and promotes complement evasion through Factor H binding. Insets: Scheme of the *Borrelia* outer membrane showing lipoprotein anchoring via a triacylated N-terminal cysteine. The proteins depicted as stylised cartoons were derived from OspA: UniProt = P0CL66; Alphafold = AF-P0CL66-F1-v4; OspC (dimer): UniProt = Q07337; Alphafold = AF-Q07337-F1-v4; CspZ: UniProt = O50665; Alphafold = AF-O50665-F1-v4.

Lyme disease (LD) remains the most prevalent vector-borne disease in the Northern Hemisphere, with over 100,000 cases reported annually in Europe, and ~476,000 in the United States [1,2]. Although LD can be treated with antibiotics, if detected early, there is currently no licensed vaccine available for humans to prevent infection. One of the key challenges in developing an effective vaccine against LD stems from the complex biology of *Borrelia* outer surface proteins (Osps), whose expression is dynamically regulated in response to distinct environmental cues, in both the arthropod vector and vertebrate host (Fig 1). Among the predominant outer surface proteins, OspC expression is induced in ticks during *Borrelia* transmission, and is sustained during early mammalian host infection, while the expression of OspA is restricted only to the tick phase [3] and is no longer expressed in *Borrelia* once the infection is established in the mammalian host [4]. As a result, infected humans only rarely develop anti-OspA antibodies [5]. Yet, OspA has proven to be a highly effective vaccine antigen, as it induces antibodies that bind to *Borrelia* within attached ticks, preventing their transmission [6]. As a new human LD vaccine candidate targeting OspA (VLA15, see below) enters Phase 3 trials, a central challenge is not only achieving protection but also sustaining it, given that its effectiveness strongly depends on maintaining high antibody titres. Serology monitoring tools may thus become an integral component of the vaccination programme, not only to distinguish between infection- and vaccine-induced antibodies, but also to track protective antibody titres over time, thereby ensuring sustained vaccine-induced immunity.

## 2. OspA-based vaccines: from a single-antigen to a global multivalent approach

The first human LD vaccine, LYMErix, targeted the full-length OspA of *Borrelia burgdorferi.* The vaccine OspA antigen sequence was derived from North American *B. burgdorferi* sensu stricto strains, offering limited coverage of the diverse *Borrelia* genospecies that cause LD in Europe (e.g., *B. afzelii*, *B. garinii*). The vaccine was later withdrawn from the market in 2002 due to public concerns and limited uptake, despite clinical validation of its safety and effectiveness [7]. These concerns arose mainly due to a potential autoimmune reaction: a segment of *B. burgdorferi* OspA shares sequence homology with human leukocyte function-associated antigen-1 (hLFA-1) within the OspA C-terminus, raising a molecular mimicry hypothesis for treatment-resistant Lyme arthritis in people carrying the HLA-DR4 antigen [8]. The second-generation LD vaccine candidate (VLA15) directly addresses both issues: it provides a broad genospecies coverage and eliminates concerns about autoimmunity.

**Fig 2:**
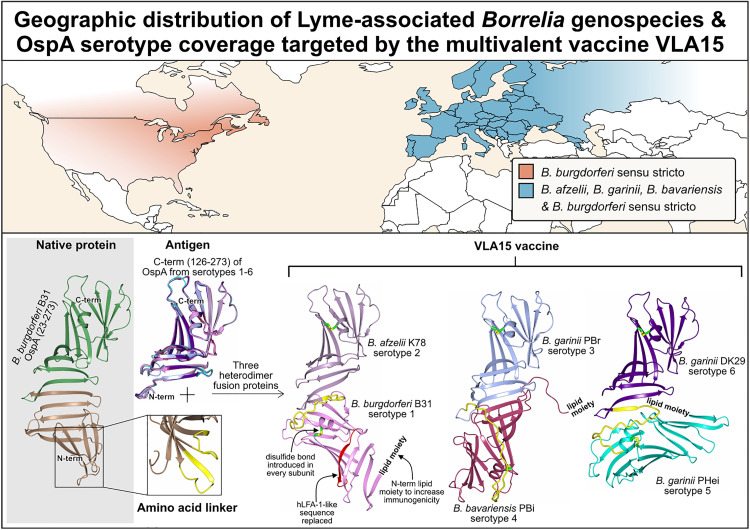
Geographic distribution of major *Borrelia* species associated with human Lyme disease and their representation in the multivalent OspA-based vaccine VLA15. The map (top panel) highlights the geographic distribution of the predominant *Borrelia* species associated with human Lyme disease across regions: *B. burgdorferi* sensu stricto in North America, and *B. afzelii*, *B. garinii*, *B. bavariensis* and *B. burgdorferi* sensu stricto in Europe and Asia. These species correspond to six predominant OspA serotypes (1–6), which underpin the multivalent OspA vaccine design and enable broad coverage across the dominant, geographically diverse *Borrelia* genospecies. The distribution map is based on the CDC’s Lyme Disease Case Map (June 2025) and LD surveillance data from Canada [32], overlayed on a base map sourced from naturalearthdata.com. The schematic (bottom panel) illustrates the VLA15 vaccine composition, which includes OspA C-terminal domains from serotypes 1–6 connected, via a 21-amino acid linker, which is derived by merging two loop regions of the N-terminal domain from serotype 1 (residues 65-GTSDKNNGSG-74 and 43-SKEKNKDGKYS-53, in which residue D53 has been mutated to S). Each subunit contains an introduced disulfide bond to increase stability. To avoid hLFA-1 mimicry, the serotype 1 hLFA-1-like epitope was replaced with the corresponding serotype 2 sequence.

VLA15, co-developed by Valneva and Pfizer, is a multivalent subunit vaccine candidate designed to provide broad protection against LD by targeting six OspA serotypes (serotypes 1–6). These serotypes represent the major *Borrelia* genospecies, causing human infection across North America and Europe [9]. To achieve broad serotype coverage, VLA15 incorporates three recombinant fusion proteins, each composed of the C-terminal OspA domains from two serotypes: serotypes 1 and 2, serotypes 3 and 4, and serotypes 5 and 6 (Fig 2). The domains within each fusion protein are connected by a 21-amino acid linker derived from two segments of the N-terminal region of OspA serotype 1 (Fig 2). Additionally, the OspA antigens in VLA15 have been optimised by introducing a single disulfide bond into every subunit to enhance structural stability. OspA proteins are natively lipidated at an N-terminal cysteine, tethering them to the plasma membrane of the spirochete [10]. In vaccine formulations, retaining this lipidation markedly enhanced immunogenicity and protective efficacy [11]. To enable this lipidation at the N-terminus of the mutant OspA C-terminal fragment heterodimers, a lipidation signal sequence derived from the *Escherichia coli* major outer membrane lipoprotein (Lpp) was introduced at their N-terminus and immediately followed C-terminally by a CSS peptide (patent publication number: WO2014006226A1). This provides the N-terminal cysteine for lipidation after signal peptide cleavage. Lastly, the hLFA-1-like fragment in OspA serotype 1 was replaced by the corresponding region from OspA serotype 2 (summarised in [12]). These designs create recombinant fusion proteins that are structurally distinct from native OspA yet retain immunological characteristics critical for vaccine effectiveness.

Like all OspA-based vaccines, VLA15 induces an unusual site of protection, taking place outside of human bodies, within the tick vector. Vaccine-induced anti-OspA antibodies from the human host would then be drained by attached ticks, as part of their blood meal, would bind its cognate antigen, neutralising *Borrelia* during tick feeding, and ultimately block transmission of *Borrelia* to humans [13]. This comes with a principal limitation of lacking an anamnestic response, leaving host immunity almost entirely dependent on sustained antibody titres rather than memory responses. The fact is also that OspA is not essential during mammalian infection [14]. In order for the vaccine to be effective, it must thus act with near-perfect efficiency at high-titre levels to sustain its capacity to neutralise spirochetes during tick feeding. If spirochetes escape neutralisation in the tick and enter the vertebrate host, OspA is downregulated and is no longer displayed on the *Borrelia* surface, leaving vaccine-induced anti-OspA antibodies without a target and making protection unlikely at that stage. This property also precludes the vaccine’s use for post-exposure prophylaxis or therapeutic intervention.

## 3. Serology-guided Lyme disease diagnosis and vaccination: unknowns and opportunities

### 3.1. Determination of protective titres

Based on the LYMErix Phase 3 trial, the CDC concluded that an anti-OspA (LA-2 antigen) antibody titre greater than 1,200 ELISA units/mL was correlated with 1-year protection against *B. burgdorferi* sensu stricto infection in the United States [15]. The breakthrough infections that occurred were mainly associated with individuals with lower titres, identifying antibody concentration as the principal correlate of vaccine efficacy. For the multivalent VLA15 vaccine (VALOR Phase 3, NCT05477524), which targets six OspA C-terminal domains, protective thresholds have not yet been defined. Because VLA15 covers multiple *Borrelia* species rather than a single U.S. strain covered by LYMErix, protective levels may need to be determined for each OspA serotype or at least for the dominant one. Notably, anti-OspA antibody levels after VLA15 vaccination decline substantially within months, particularly in elderly individuals, who often generate lower and shorter-lived titres [16,17]. In Europe, where *Borrelia* isolates causing breakthrough infections are rarely genotyped, such thresholds will require further refinement. Establishing serotype-specific minimal protective titres could improve serology-guided booster scheduling and support data-driven vaccine optimisation.

### 3.2. Impact of VLA15 on current LD diagnosis and opportunities for future precision

**Fig 3:**
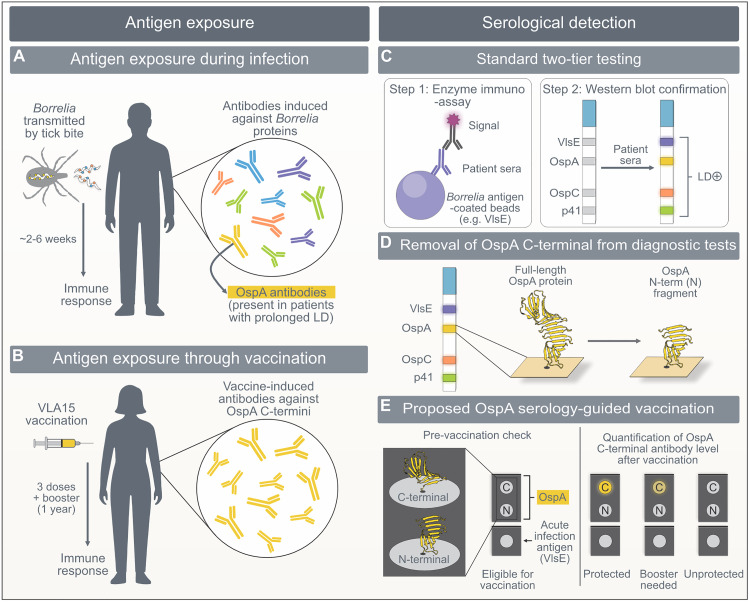
Schematic overview of current and emerging opportunities in Lyme disease (LD) diagnostics and serology-guided vaccination. A) During mammalian infection, *Borrelia burgdorferi* sensu lato (s.l.) expresses surface proteins that elicit a specific humoral immune response. Commonly targeted antigens include VlsE, OspC, and flagellin (p41), whereas antibodies against OspA are rarely detected (≤15% of cases). B) The VLA15 vaccine is composed only of the C-termini of OspA of 6 *B. burgdorferi* s.l. serotypes. C) The current standard two-tier testing for LD typically combines an enzyme immunoassay (EIA) using a mixture of selected antigens or a single-antigen (e.g., VlsE) with a confirmatory strip-blot assay detecting IgG and IgM antibodies against multiple *Borrelia* antigens. D) These strip blots often contain full-length OspA antigens, which will also detect antibodies in vaccinated individuals following VLA15 implementation. E) Diagnostic immunostrips can be multiplexed using microchip-based technologies, which can provide more targeted and detailed information by distinguishing infection-induced from vaccine-induced antibody responses. Provided a vaccinated person is sero-negative (grey) for acute (e.g., VlsE) or prolonged LD (N-terminal OspA) infection, such individual will be possibly eligible for VLA15 vaccination. These technologies can also enable quantitative, time-resolved tracking of vaccine-induced antibody levels (C-terminal OspA, yellow). Signal intensity will either indicate the anticipated protective level or significant waning of vaccine induced antibody titres, indicating a call for a vaccine booster. Inclusion of acute infection antigens (e.g., VlsE) in these multiplex immunoassays will also allow to detect breakthrough infections. The correlation between circulating antibody levels and the occurrence of LD infections will help define the precise threshold of protective immunity.

The fact that VLA15 includes only the C-termini of OspA proteins, and that anti-OspA-antibodies are rare in sera as a response to LD infection [18], allows tracking levels of vaccine-induced antibodies. Even though the whole OspA protein is immunogenic and can, in rare cases, be exposed during infection to host immunity, hallmarking, e.g., prolonged arthritis [19], the presence of anti-OspA IgG antibodies in sera will be mostly indicative of vaccine-induced antibodies in vaccinated individuals. We recommend revising current diagnostic antigen sets, which typically contain full-length OspA (frequently derived from multiple *Borrelia* strains in Europe), to instead include distinct N- and C-terminal fragments of OspA (Fig 3). Such antigenic microchip, for example, would contain: (1) established antigens to flag past or present LD [20], (2) the N-terminus of OspA to detect the rare anti-OspA responses observed in prolonged episodes of LD (thereby identifying individuals for whom OspA-based vaccination should not be recommended), and (3) the C-terminus of OspA that would monitor the presence of vaccine-induced antibodies for people with negative N-terminus OspA immunodetection (Fig 3). For quantitative readouts, titration of OspA C-termini would allow tracking of post-vaccination levels of antibodies (Fig 3). Once validated correlates of protection are established, this approach could guide seasonal vaccine strategies and ensure protection during peak tick activity.

This opportunity currently applies only to the VLA15 vaccine. The mRNA vaccines mRNA-1975 and mRNA-1982 developed by ModernaTX, Inc. (ClinicalTrials.gov: ID NCT05975099), code for full-length proteins (WO2024215721A1) that will prevent any potential opportunity for differentiation of vaccine-induced and rare infection-induced anti-OspA IgG antibodies in the sera. It is important to realise that with the introduction of the OspA-based VLA15 vaccine, modified two-tier testing (MTTT) currently using whole cell lysate [21] will also become susceptible to vaccine-induced seroreactivity in uninfected patients. Therefore, platforms with informative single-antigens [22] or single-epitopes [23,24] may offer a more practical tool, with detailed precision, for the diagnosis of infections and determination of vaccine titres.

### 3.3. Lyme disease vaccination eligibility

Many *Borrelia* infections in endemic regions remain subclinical and untreated, resulting in seropositive individuals without a documented history of LD. While there is currently no publicly available efficacy or safety data for VLA15 in individuals with a history of LD, it is reasonable to expect that future studies or post-licensure monitoring will address this gap. To minimise safety risks and ensure that immunogenicity data reflect naïve populations, phase 1 [16,25–27], phase 2 [16,26,27], and the ongoing phase 3 (ClinicalTrials.gov ID: NCT05477524) clinical trials of the VLA15 vaccine have excluded participants with detectable anti-*Borrelia* antibodies, as well as individuals with autoimmune conditions or inflammatory arthritis. Although no vaccine-associated adverse effects have been reported in seronegative individuals enrolled in these trials, theoretical concerns remain regarding possible inflammatory responses in previously Lyme-exposed individuals. This is likely due to potential immune recognition of residual OspA antigen in immune-privileged sites, such as synovial tissue. Therefore, more data need to be collected on the vaccination of people with a history of LD, or pre-vaccination immune-screening, using conventional or emerging serological tools in future clinical practice to identify individuals with prior exposure.

## 4. Beyond OspA: is there a way for multiprotein vaccines?

The inclusion of *Borrelia* antigens expressed during early human infection offers a promising avenue for the design of multi-antigen LD vaccines. Such strategies could synergistically combine OspA-based transmission-blocking components with antigens targeting transmitted *Borrelia* that breached anti-OspA protection. OspC emerges as a promising candidate due to its crucial role during the initial phase of mammalian infection [28]. OspC is markedly upregulated upon spirochete transmission to the vertebrate host, facilitating successful colonisation and eliciting a robust antibody response. However, its utility in vaccine development is complicated by significant antigenic variability, with numerous OspC serotypes circulating in endemic *Borrelia* populations. To address this diversity, “chimeritope” vaccines have already been successfully developed for canines. These fusion proteins incorporate immunogenic epitopes from multiple OspC variants into a single, mosaic antigen. The canine Lyme vaccine, VANGUARDcrLyme (Zoetis), exemplifies this approach by including a chimeric OspC antigen (Ch14), derived from segments of 14 distinct OspC types, alongside OspA. This strategy induces antibodies with broad reactivity, providing extensive protection [29]. Murine models have confirmed that OspC chimeritopes similarly generate cross-protective antibody responses, emphasising their potential translational relevance to human vaccines [29]. Critically, incorporating OspC, or other antigens expressed early during host infection into OspA-based vaccines, may enable a rapid anamnestic response to these antigens upon spirochete entry into the host.

Another promising vaccine candidate is the complement regulator-acquiring surface protein 2 (also known as CRASP-2 or CspZ). Although CspZ is known to bind the complement regulator factor H (FH), it thus blocks activation of complement on the surface of the spirochete. CspZ-YA, a double mutant (two-point mutations) of CspZ from *B. burgdorferi*, has been engineered to lack its native FH-binding activity, thereby exposing epitopes that are otherwise shielded by FH. By further introducing protein-stabilising mutations into CspZ-YA, namely I183Y or C187S, a robust bactericidal antibody response is observed, which protects against infection in mouse models [30]. These results suggest that either CspZ-YA_C187S_ or CspZ-YA_I183Y_ would be promising components for inclusion into future multivalent LD vaccines. It should be noted that while these antigens (e.g., OspC, CspZ) show strong potential in preclinical studies, to date, no multi-antigen vaccines have entered human clinical trials; all clinical-stage candidates remain OspA-based.

## 5. Summary and outlook

Lyme disease prevention is entering a transformative phase. The VLA15 vaccine represents the most advanced candidate to date, overcoming historical limitations by broadening genospecies coverage and resolving safety concerns. Yet, the effectiveness of OspA-based vaccines depends fundamentally on maintaining high antibody titres at the time of tick exposure. Emerging miniaturised high-throughput serological microarray platforms, designed to differentiate infection- and vaccine-induced antibodies, could not only enable robust multi-epitope LD diagnosis but also incorporate epitopes for monitoring vaccine-induced titres. Such data-based booster scheduling may help sustain protective immunity through each tick season [31], preventing breakthrough infections. However, defining human correlates of protection remains a key unmet need. With most technical limitations in vaccine design now resolved, the integration of data-guided, personalised serology may enhance the likelihood of success for the second generation of vaccine strategies capable of preventing Lyme disease in humans.
